# Cost-Effectiveness of Pharmacist Prescribing for Managing Hypertension in the United States

**DOI:** 10.1001/jamanetworkopen.2023.41408

**Published:** 2023-11-03

**Authors:** Dave L. Dixon, Karissa Johnston, Julie Patterson, Carlo A. Marra, Ross T. Tsuyuki

**Affiliations:** 1 Department of Pharmacotherapy and Outcomes Science, Center for Pharmacy Practice Innovation, Virginia Commonwealth University School of Pharmacy, Richmond; 2 Broadstreet Health Economics and Outcomes Research, Vancouver, British Columbia, Canada; 3 School of Pharmacy, University of Otago, Dunedin, New Zealand; 4 Department of Medicine (Cardiology), Faculty of Medicine and Dentistry, University of Alberta, Edmonton, Alberta, Canada

## Abstract

**Importance:**

Pharmacist-led interventions can significantly improve blood pressure (BP) control. The long-term cost-effectiveness of pharmacist-prescribing interventions implemented on a large scale in the US remains unclear.

**Objective:**

To estimate the cost-effectiveness of implementing a pharmacist-prescribing intervention to improve BP control in the US.

**Design, Setting, and Participants:**

This economic evaluation included a 5-state Markov model based on the pharmacist-prescribing intervention used in The Alberta Clinical Trial in Optimizing Hypertension (or RxACTION) (2009 to 2013). In the trial, control group patients received an active intervention, including a BP wallet card, education, and usual care. Data were analyzed from January to June 2023.

**Main Outcomes and Measures:**

Cardiovascular (CV) events, end-stage kidney disease events, life years, quality-adjusted life years (QALYs), lifetime costs, and lifetime incremental cost-effectiveness ratio (ICER). CV risk was calculated using Framingham risk equations. Costs were based on the reimbursement rate for level 1 encounters, medication costs from published literature, and event costs from national surveys and pricing data sets. Quality of life was determined using a published catalog of EQ-5D utility values. One-way sensitivity analyses were used to assess alternative reimbursement values, a reduced time horizon of 5 years, alternative assumptions for BP reduction, and the assumption of no benefit to the intervention after 10 years. The model was expanded to the US population to estimate population-level cost and health impacts.

**Results:**

Assumed demographics were mean (SD) age, 64 (12.5) years, 121 (49%) male, and a mean (SD) baseline BP of 150/84 (13.9/11.5) mm Hg. Over a 30-year time horizon, the pharmacist-prescribing intervention yielded 2100 fewer cases of CV disease and 8 fewer cases of kidney disease per 10 000 patients. The intervention was also associated with 0.34 (2.5th-97.5th percentiles, 0.23-0.45) additional life years and 0.62 (2.5th-97.5th percentiles, 0.53-0.73) additional QALYs. The cost savings were $10 162 (2.5th-97.5th percentiles, $6636-$13 581) per person due to fewer CV events with the pharmacist-prescribing intervention, even after the cost of the visits and medication adjustments. The intervention continued to produce benefits in more conservative analyses despite increased costs as the ICER ranged from $2093 to $24 076. At the population level, a 50% intervention uptake was associated with a $1.137 trillion in cost savings and would save an estimated 30.2 million life years over 30 years.

**Conclusion and Relevance:**

These findings suggest that a pharmacist-prescribing intervention to improve BP control may provide high economic value. The necessary tools and resources are readily available to implement pharmacist-prescribing interventions across the US; however, reimbursement limitations remain a barrier.

## Key Points

**Question:**

What would be the cost-effectiveness of implementing a pharmacist-prescribing intervention to improve blood pressure control in the United States?

**Findings:**

In this simulated cost-effectiveness analysis of a 5-state Markov model, 50% uptake of a pharmacist-prescribing intervention to improve blood pressure control was associated with a $1.137 trillion in cost savings and could save an estimated 30.2 million life years over 30 years.

**Meaning:**

These findings suggest that pharmacist-prescribing interventions to improve blood pressure control would provide high economic value compared with usual care.

## Teaser

This economic evaluation estimates the cost-effectiveness of implementing a pharmacist-prescribing intervention to improve blood pressure control in the US.

## Introduction

Hypertension (HTN) is the leading preventable cause of death and disability throughout the world.^1^ More than 100 million people in the US have HTN, a significant risk factor for the development of cardiovascular disease (CVD) and kidney disease.^2^ Health care costs associated with HTN in the US alone exceeded $130 billion between 2003 and 2014.^3^ Despite affordable medications and lifestyle interventions proven to reduce blood pressure (BP), BP control rates in the US are declining.^4^ Currently, only 1 in 4 adults with HTN has their BP under control (ie, less than 130/80 mm Hg).^2^

In 2020, the US Surgeon General issued a Call to Action to Control Hypertension,^5^ which “seeks to avert the negative health effects of HTN across the US by identifying interventions that can be implemented, adapted, and expanded across diverse settings.” The goals include making HTN a national priority; ensuring the places where people live, learn, work, and play support HTN control; and optimizing patient care for HTN. One of the primary strategies promotes standardized treatment approaches and guideline-recommended care with an emphasis on team-based care.^5^

Pharmacists are well placed in the community to screen and manage HTN because they see patients up to 10 times more frequently than physicians.^6^ Numerous randomized clinical trials^7,8,9,10^ of pharmacist-led case-finding and prescribing interventions have improved HTN outcomes. Given this evidence and the compelling need for new solutions to reduce the clinical and economic burden of uncontrolled HTN, we conducted a cost-effectiveness analysis of implementing pharmacist prescribing for HTN management in the US.

## Methods

This economic evaluation followed the Consolidated Health Economic Evaluation Reporting Standards (CHEERS) reporting guideline. Per the Common Rule, institutional review and informed consent were not required because this research did not involve human participants.

**Figure 1.**
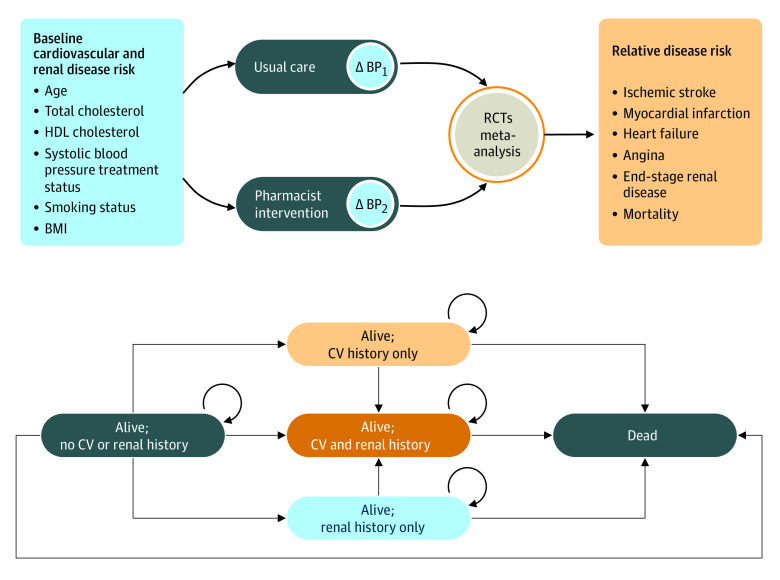
Model Structure Abbreviations: BMI, body mass index (calculated as weight in kilograms divided by height in meters squared); BP, blood pressure; CV, cardiovascular; HDL, high-density lipoprotein; RCT, randomized controlled trial.

A pharmacoeconomic model was developed in Microsoft Excel to assess the potential impact of pharmacist prescribing for HTN compared with usual care (status quo) on long-term costs and health outcomes in the US. The implementation of the model for a Canadian population has been previously described in greater detail^11^; this structure was used and updated to reflect the US population and health care system. Briefly, the model was structured as a 5-state Markov model, with patients entering the model with uncontrolled HTN and no additional history of cardiovascular (CV) or kidney disease. Over time, patients were at risk of developing CV and/or kidney disease and subsequent death (Figure 1). All patients were at risk for all-cause mortality based on general population life tables, with an increased risk of mortality in individuals following a CV event. The conceptual model assumed that the pharmacist-prescribing intervention would reduce BP, with a resultant decreased risk of CV and kidney disease; the costs of implementing pharmacist-prescribing HTN management were thus compared with long-term cost offsets as well as health and mortality benefits resulting from this BP reduction.

The base case scenario was a third-party payer perspective, with a 30-year time horizon, 1-year model cycles, and costs and quality-adjusted life years (QALYs) discounted at 3% per annum.^12^ Results are reported at both the individual level and scaled up to the US population based on the number of individuals with uncontrolled HTN.

### Clinical Model Structure

The Alberta Clinical Trial in Optimizing Hypertension (R_x_ACTION) was conducted in Alberta, Canada from 2009 to 2013.^10^ This analysis was conducted in 2023 and used a model base case based on the mean 6-month reduction in systolic BP (SBP) (−18.3 mm Hg) observed with the pharmacist intervention in the R_x_ACTION study, which involved pharmacist assessment and counseling of BP, antihypertensive medication review, and prescribing antihypertensives in a face-to-face encounter. Pharmacist follow-up occurred every 4 weeks until BP was at goal for 2 consecutive visits followed by 12-week intervals for the remainder of the 24-week study duration. In the model, it was assumed that this would correspond to 6 visits in the first year and quarterly visits thereafter. For the comparator group, we assumed that BP would remain at baseline levels. We did not use the control group from the R_x_ACTION trial because it was an active intervention. Baseline clinical and demographic characteristics were based on the trial population (eTable 1 in Supplement 1).

The risk of CVD over time for the control arm was calculated based on Framingham risk equations for myocardial infarction (MI), stroke, heart failure (HF), and angina given baseline BP levels.^13,14,15^ The association between SBP reduction in the intervention group and reduced risk of CVD was estimated using results from the Blood Pressure Lowering Treatment Trialists’ Collaboration.^16^ A regression analysis was conducted based on the reported values for SBP and risk reduction of major CV events, and the resulting slope was used to estimate the impact of a 1-unit reduction on the relative risk. The resulting estimated association was a 0.026 (SE, 0.004) decrease in relative risk of CVD per each mm Hg decrease in SBP.

The impact of BP on kidney disease was characterized by the risk of end-stage kidney disease (ESKD), which was based on a reported association between BP categories and ESKD incidence observed in a US historical cohort study and a 25-year follow-up study.^17,18^ Rates per 100 000 person-years were reported by category (normal, pre-HTN, stage 1 HTN, and stage 2 HTN), and converted to annual probabilities. For the modeled population at baseline, the mean BP corresponded to stage 1 HTN, which corresponded to an ESKD rate of 19.5 per 100 000 person-years, or an annual risk of 0.000194 per person. This annual risk was retained for the control arm. For the treatment arm, regression analysis of risk by BP category was conducted to estimate a risk reduction of 0.77 associated with observed BP reduction, which was applied to result in an annual ESKD probability of 0.000150 for the pharmacist-prescribing intervention group. Mortality was based on US life tables, with a hazard ratio of 1.71 applied to account for the increased risk of mortality in a population with CVD.^19^

### Costs

In the base case of the model, all pharmacist assessments were assumed to incur a cost of $23.10, reflecting the 2019 reimbursement rate for Current Procedural Terminology (CPT) 99211 (level 1 patient encounters).^20^ Visits were assumed to be monthly for the first 3 months (assumed time until HTN became controlled), followed by quarterly, with 6 pharmacist visits in the first year and 4 annually after that. Given that the clinical model included pharmacist prescribing of medications, we assumed that patients receiving the intervention would incur an incremental medication cost of $32.78/mo, based on the mean monthly medication cost for individuals with HTN in the US. This was chosen conservatively to maximize the cost of the pharmacist intervention; the true incremental medication cost is likely lower given that some usual care patients receive physician-prescribed HTN medications, and pharmacist-prescribing interventions often result in discontinuation of less appropriate or effective medications.^10,21^ Annual background all-cause health care costs for all individuals were based on age-specific values reported by the Agency for Healthcare Research and Quality.^22^

For individuals experiencing health events, the cost of the event was stratified into the first-year postevent and subsequent years. Costs for CV events (ie, stroke, heart failure, angina, and MI) were based on reported values from a US microsimulation model of HTN screening strategies, which used Medical Expenditure Panel Survey data. The cost for ESKD was based on US Renal Data System data (eTable 2 in Supplement 1).^23^ All costs were inflated to 2021 US dollar based on the US Consumer Price Index-Medical Care.^24^

### Health-Related Quality of Life

Health state utilities were taken from a published catalog of EQ-5D utility values in the US. Baseline utilities were 0.867 for patients without ESKD or CVD and age-adjusted using a utility decrement of 0.00029 per year after age 70 years.^25^ The utility values included in the model were 0.694 for stroke, 0.725 for MI, 0.636 for HF, 0.709 for angina, and 0.708 for ESKD. Disease-specific utilities were assumed to be chronic and continued to apply years after the event.

### Sensitivity Analyses

One-way sensitivity analyses were used to examine the impact of variation in key inputs, including (1) increased costs per pharmacist visit, reflecting reimbursement values aligned with a greater likelihood of dissemination and sustainability—$100 for an initial visit and $50 per follow-up; (2) reduced time horizon to 5 years; (3) alternative assumptions regarding SBP decrease, ranging from −5 to −27 mm Hg; (4) examining each type of health benefit (ie, reductions in stroke, MI, angina, HF, and ESKD) in isolation; (5) assuming that the HTN benefit is only sustained for 10 years, after which point there is no benefit to the intervention; and (6) a conservative scenario in which the BP decrease is assumed to be −10 mm Hg, losing 50% of benefit at 5 years, and 100% of benefit at 10 years. The range of BP values explored in sensitivity analysis reflects existing literature on the effect of pharmacist interventions on BP. A meta-analysis^7^ reported that pharmacist interventions decreased mean SBP by an additional −7.6 mm Hg compared with usual care, but the types of pharmacist interventions in the included studies were heterogeneous and did not include prescriptive authority. Alternatively, the cluster-randomized trial of a pharmacist-prescribing intervention in black barbershops reported a mean reduction in SBP of −27 mm Hg in the intervention group; thus our use of −18.3 mm Hg from the R_x_ACTION is reasonable.

In addition to the 1-way sensitivity analyses, a 1000-iteration probabilistic sensitivity analysis was conducted to reflect the impact of stochastic parameter uncertainty on results. This included probabilistic variability of cost, clinical, and health-related quality of life parameters, including the SBP reduction and the relationship between SBP and clinical event risk.

### Epidemiologic Analyses

Base case cost-effectiveness results were expanded to the US population to estimate cumulative cost and health impacts over 30 years. Individual-level results output by the model were multiplied year-over-year by the estimated number of incidents and prevalent patients with uncontrolled HTN assumed to be accessing the intervention. This time horizon was chosen to capture the lifetime of the model cohort. The prevalence of uncontrolled HTN was estimated to be 92.1 million^26^; it was assumed that 50% of eligible individuals would access the intervention. Over a 30-year time horizon, incident cases of HTN were added each year based on a US cohort study.^27^ It was assumed that the 50% rate of intervention use would persist among incident cases. Clinical and cost outcomes were assessed over the time horizon.

## Results

Briefly, the R_x_ACTION trial enrolled 248 participants (mean [SD] age, 64 [12.5] years; 121 [49%] male; 41 [15%] currently smoked; and 109 [48%] had diabetes). The mean (SD) baseline BP was 150/84 (13.9/11.5) mm Hg with a mean (SD) of 1.7 (1.2) antihypertensives per participant. The pharmacist intervention achieved a significant reduction in SBP at 6 months compared with the active control group (−18.3 mm Hg vs −11.8 mm Hg, respectively; *P* < .001).

**Figure 2.**
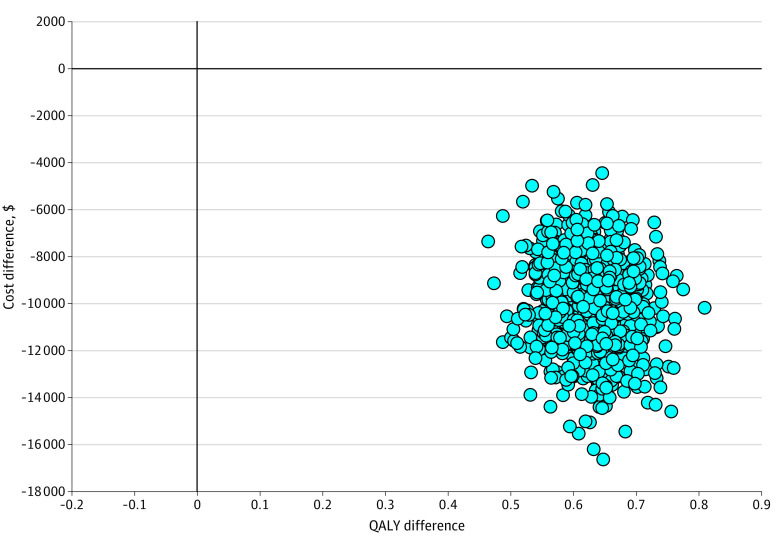
Probabilistic Sensitivity Analysis of Pharmacist Intervention Abbreviation: QALY, quality-adjusted life year.

In the base case analysis over a 30-year time horizon, the pharmacist intervention was associated with 2100 fewer cases of CVD and 8 fewer cases of kidney disease per 10 000 patients. Per patient, the intervention was associated with 0.34 additional life years (discounted) and 0.62 additional QALYs (discounted) (Table 1). The intervention also resulted in overall cost savings of $10 162 per person, as the cost reduction associated with fewer CV events more than offset the cost of pharmacist visits and medication adjustments (Table 1). When comparing health care costs only (ie, excluding the costs of the intervention itself) mean costs were $189 648 in the control group and $172 167 in the intervention group, for a savings of $17 481. As the pharmacist-prescribing intervention was associated with both better health outcomes and lower costs, it was found to be dominant (discounted and undiscounted). Results were robust in the probabilistic sensitivity analysis, because 100% of probabilistic iterations were in the economically dominant quadrant of the cost-utility plane (Figure 2).

**Table 1.** Base Case, Quality-Adjusted Life Years, and Costs^a^

Characteristic	Usual care	Pharmacist intervention	Difference
Base case			
Cardiovascular events	0.61	0.40	−0.21
End-stage kidney disease events	0.0038	0.0030	−0.0008
Life years			
Discounted	14.6 (14.3 to 14.9)	15.0 (14.8 to 15.2)	0.34 (0.23 to 0.45)
Undiscounted	19.7	20.3	0.63
Quality-adjusted life years			
Discounted	11.8 (11.6 to 12.0)	12.4 (12.3 to 12.6)	0.62 (0.53 to 0.73)
Undiscounted	15.7	16.7	1.03
Costs			
Discounted	$189 648 ($151 188 to $237 055)	$179 485 ($140 586 to $225 972)	−$10 162 (−$13 581 to −$6636)
Undiscounted	$276 218	$262 593	−$13 625
Category-specific costs (discounted)			
Intervention costs	$0	$7318	$7318
Background medical costs	$97 481	$99 751	$2270
Total cardiovascular disease	$45 506	$28 242	−$17 264
Stroke	$10 652	$6595	−$4057
Myocardial infarction	$17 905	$11 152	−$6753
Angina	$5631	$3493	−$2138
Heart failure	$11 319	$7003	−$4317
Chronic kidney disease	$46 661	$44 174	−$2487

^a^ Results presented are deterministic. Where available for key base-case outcomes, 2.5th to 97.5th percentiles from the probabilistic sensitivity analysis are included.

In 1-way sensitivity analyses, results remained dominant when pharmacist costs were increased from the CPT level 1 reimbursement rate of $23.10 to $100 for an initial visit and $50 per follow-up visit, indicating that further incentivizing the pharmacist intervention would not jeopardize the resulting value of the service and would offset the pharmacist labor costs (Table 2). The intervention also continued to dominate usual care when benefits were only accrued for 10 years, at which point the intervention was assumed to be equivalent to usual care. Although in this scenario, cost savings were reduced to $5744 and QALY benefits were reduced to 0.08 per patient. This was further reduced to cost savings of $521 in a scenario where the SBP reduction was reduced to −10 mm Hg, with 50% efficacy loss at 5 years and 100% efficacy at 10 years. However, economic dominance was still retained. In a series of more conservative analyses (ie, reduced effectiveness of a less-intensive intervention, considering each respective health outcome in isolation), the intervention continued to result in health benefits, but with an increase in costs; incremental cost-effectiveness ratios ranged from $2093 to $24 076, well within standard thresholds for cost-effectiveness (Table 2). Reducing the time horizon to 5 years yielded an incremental cost-effectiveness ratio of $16 987.

**Table 2.** Incremental Cost-Effectiveness Ratio and One-Way Sensitivity Analyses^a^

One-way sensitivity analyses	ICER, $
Increased cost per pharmacist visit ($100 first followed by $50)	Intervention dominates
5-y Time horizon	16 987
Systolic blood pressure reduction: 7.6 mm HG	2093
Systolic blood pressure reduction: 27 mm HG	Intervention dominates
Only stroke benefits included	14 572
Only myocardial infarction benefits included	6548
Only angina benefits included	21 995
Only heart failure benefits included	14 895
Only kidney benefits included	24 076
Attenuating benefits to 10 y	Intervention dominates
Systolic blood pressure reduction: 10 mm HG, attenuating benefits to 10 y with 50% efficacy reduction after 5 y	Intervention dominates

Abbreviation: ICER, incremental cost-effectiveness ratio.

^a^ Results presented are deterministic. Where available for key base-case outcomes, 2.5th to 97.5th percentiles from the probabilistic sensitivity analysis are included.

**Figure 3.**
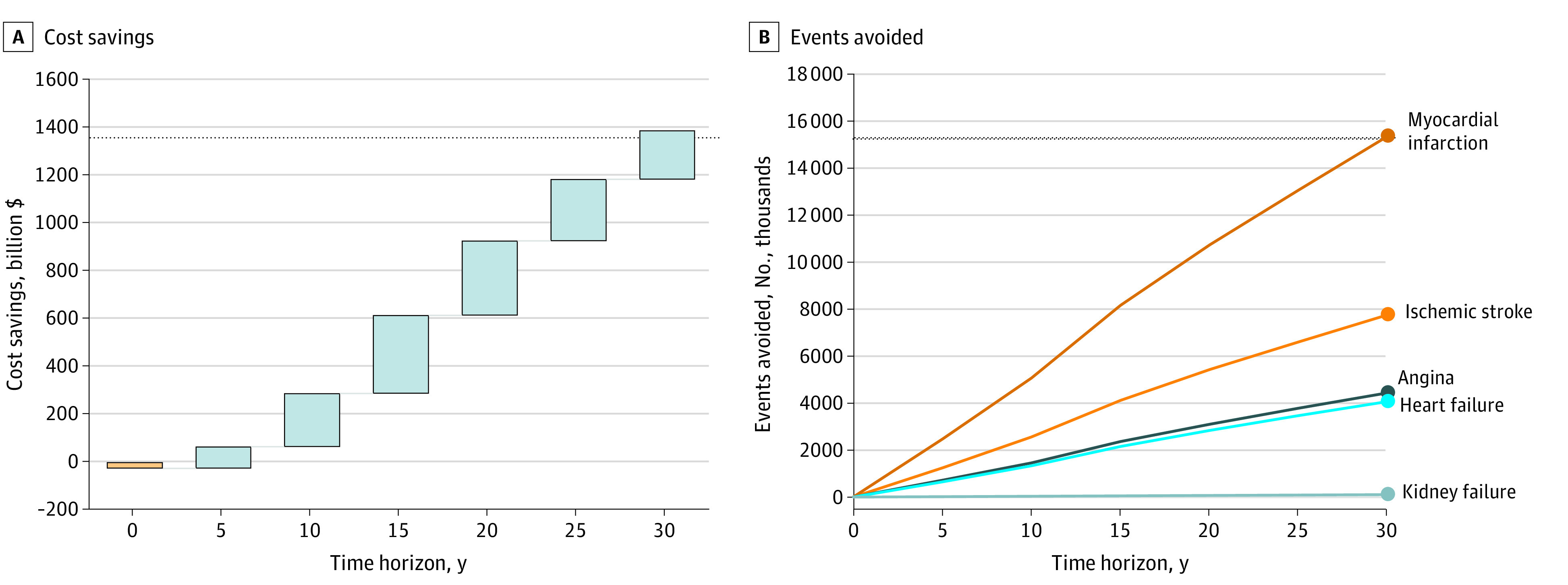
Cumulative Cost Savings and Health Outcomes Averted With Estimated Population Use of the Pharmacist Intervention

In a more comprehensive 1-way assessment of the association between incremental costs and QALYs across a range of SBP values, the pharmacist intervention was associated with increased QALYs and was associated with reduced costs for SBP reduction of −9 mm Hg or greater (eFigure 1 in Supplement 1). For a hypothetical SBP reduction between −5 and −9 mm Hg, although costs were greater for the pharmacist intervention, incremental cost-effectiveness ratios remained at cost-effective levels, ranging from $500 to $16 000. When the model with base case settings was expanded to the population level, it was estimated that with a 50% access rate, the pharmacist intervention would lead to $1.137 trillion in cost savings and save 30.2 million life years over 30 years (Figure 3).

## Discussion

Pharmacist interventions significantly improve BP control,^7^ but the economic impact of widespread adoption of such interventions has been unclear. Our study demonstrates that a pharmacist prescribing intervention would save $10 162 per person over a 30-year time horizon with and at the population level, a cumulative savings of $1.13 trillion dollars. These savings were largely attributable to a reduction in CV events due to improved BP control with the intervention. These findings mirror those from a similar analysis evaluating the implementation of this model in Canada.^11^ The cost savings in that study were less at $6364 per person, translating to a population benefit of 15.7 billion over 30 years, likely due to the lower overall health care costs in Canada compared with the US.

There is a critical need for innovative approaches, such as pharmacist-led interventions, to improve BP control. Between 2010 and 2019, there was a 23.1% increase in HTN-related mortality in the US.^28^ In 2019, the rate of HTN-related death among Black individuals aged 35 to 64 years was 96.3 events per 100 000—the highest of any race or ethnicity. Importantly, pharmacist-led interventions have been shown to significantly improve BP control among Black individuals and individuals of racial and ethnic minoritized groups.^8,29,30,31^ There is also a sense of urgency for broader implementation of pharmacist interventions to improve BP control given the worsening shortage of primary care clinicians, which could reach between 17 800 and 48 000 by the year 2034.^32^ Given that 95% of individuals in the US live within 5 miles of a pharmacy, pharmacists are a possible solution to improve care access.^33^

Widespread implementation of pharmacist-prescribing interventions targeting uncontrolled HTN is feasible but will require continued advancement in pharmacist scope of practice legislation and eligibility for reimbursement through the Centers for Medicare & Medicaid Services. Today, 49 states and the District of Columbia have legislative provisions allowing pharmacist prescriptive authority through collaborative practice agreements, standing orders, or statewide protocols.^34^ Such collaborative models often occur between pharmacists and physicians and permit prescriptive authority to pharmacists to initiate, adjust, or discontinue medications for specific medical conditions per an agreed-upon protocol or current clinical practice guidelines.^35^ This approach is also evidence-based as it has been used in randomized trials demonstrating the effectiveness of physician-pharmacist collaborative models for HTN.^8,30^ Expansion of prescriptive authority for pharmacists could increase access for those with limited or no source of primary care, which disproportionately affects males, underrepresented minorities, the uninsured, and those living in the southern US.^36^

While pharmacists may participate in collaborative models, pharmacists are infrequently recognized by payers because they are not recognized clinicians under the Social Security Act. Pharmacists can bill for services incident to those provided by a physician or advanced practice clinician; however, this is limited to Level 1, which is only $23.10 for 5 minutes of clinical services and insufficient for the level of service provided.^37^ Our analysis showed that a pharmacist-prescribing intervention would remain cost-effective if pharmacists received a hypothetical reimbursement of $100 for the initial visit and $50 for each follow-up. While some states have recently passed clinician status legislation, much work remains to ensure pharmacists are adequately compensated for the clinical services they provide.

### Limitations

This study had limitations. The cost savings assume a 50% uptake of the intervention, and the savings magnitude depends on uptake. However, if pharmacists are appropriately incentivized through adequate reimbursement for providing the service, this level of uptake is likely an underestimate. Another assumption is that BP control did not change in the comparator group, and a proportion of patients in the comparator group may have improved BP control with usual care. Further, the proportion of patients with uncontrolled HTN continues to rise and has only worsened because of the COVID-19 pandemic. These findings cannot be generalized to other populations with HTN (eg, pregnancy), and we were unable to determine how alternative delivery methods (eg, telehealth) would impact the cost-effectiveness of this model.

## Conclusions

This economic analysis suggests that pharmacist-prescribing interventions are cost-effective, result in significant estimated savings for the health care system, and are economically dominant. Assuming a 50% adoption rate, pharmacist-prescribing interventions would save an estimated $10 162 per person over a 30-year time horizon with cumulative population-level savings of more than a trillion dollars. The necessary tools (eg, collaborative practice, treatment algorithms) and resources (eg, patient access to community pharmacies) are readily available to implement pharmacist-prescribing interventions across the US; however, reimbursement limitations remain a barrier.

## Notes

Supplement 1. **eTable 1.** Assumed Patient Characteristics for Pharmacist Hypertension Intervention, Based on Observed Population in Tsuyuki et al^10^ Clinical Trial **eTable 2.** Model US Cost Inputs **eFigure 1.** Association Between Cumulative Cost Savings and QALY Benefit Across a Range of Hypothetical SBP Reductions Associated With the Pharmacist Intervention

Supplement 2. Data Sharing Statement

## References

[1] Mills KT, Stefanescu A, He J. The global epidemiology of hypertension. Nat Rev Nephrol. 2020;16(4):223-237. doi:10.1038/s41581-019-0244-2 32024986 PMC7998524

[2] Facts about hypertension. Centers for Disease Prevention and Control. Accessed January 26, 2021. https://www.cdc.gov/bloodpressure/facts.htm

[3] Kirkland EB, Heincelman M, Bishu KG, . Trends in healthcare expenditures among US Adults with hypertension: national estimates, 2003-2014. J Am Heart Assoc. 2018;7(11):e008731. doi:10.1161/JAHA.118.008731 29848493 PMC6015342

[4] Muntner P, Hardy ST, Fine LJ, . Trends in Blood Pressure Control Among US Adults With Hypertension, 1999-2000 to 2017-2018. JAMA. 2020;324(12):1190-1200. doi:10.1001/jama.2020.14545 32902588 PMC7489367

[5] US Department of Health and Human Services. The Surgeon General’s Call to Action to Control Hypertension. Accessed November 5, 2020. https://www.hhs.gov/sites/default/files/call-to-action-to-control-hypertension.pdf

[6] Tsuyuki RT, Beahm NP, Okada H, Al Hamarneh YN. Pharmacists as accessible primary health care providers: Review of the evidence. Can Pharm J (Ott). 2018;151(1):4-5. doi:10.1177/1715163517745517 29317929 PMC5755826

[7] Santschi V, Chiolero A, Colosimo AL, . Improving blood pressure control through pharmacist interventions: a meta-analysis of randomized controlled trials. J Am Heart Assoc. 2014;3(2):e000718-e000718. doi:10.1161/JAHA.113.000718 24721801 PMC4187511

[8] Victor RG, Lynch K, Li N, . A cluster-randomized trial of blood-pressure reduction in black barbershops. N Engl J Med. 2018;378(14):1291-1301. doi:10.1056/NEJMoa1717250 29527973 PMC6018053

[9] Tsuyuki RT, Al Hamarneh YN, Jones CA, Hemmelgarn BR. The effectiveness of pharmacist interventions on cardiovascular risk: the multicenter randomized controlled RxEACH trial. J Am Coll Cardiol. 2016;67(24):2846-2854. doi:10.1016/j.jacc.2016.03.528 27058907

[10] Tsuyuki RT, Houle SK, Charrois TL, ; RxACTION Investigators. Randomized trial of the effect of pharmacist prescribing on improving blood pressure in the community: the alberta clinical trial in optimizing hypertension (RxACTION). Circulation. 2015;132(2):93-100. doi:10.1161/CIRCULATIONAHA.115.015464 26063762

[11] Marra C, Johnston K, Santschi V, Tsuyuki RT. Cost-effectiveness of pharmacist care for managing hypertension in Canada. Can Pharm J (Ott). 2017;150(3):184-197. doi:10.1177/1715163517701109 28507654 PMC5415065

[12] Sanders GD, Neumann PJ, Basu A, . Recommendations for conduct, methodological practices, and reporting of cost-effectiveness analyses: second panel on cost-effectiveness in health and medicine. JAMA. 2016;316(10):1093-1103. doi:10.1001/jama.2016.12195 27623463

[13] D’Agostino RB Sr, Vasan RS, Pencina MJ, . General cardiovascular risk profile for use in primary care: the Framingham Heart Study. Circulation. 2008;117(6):743-753. doi:10.1161/CIRCULATIONAHA.107.699579 18212285

[14] Pencina MJ, D’Agostino RB Sr, Larson MG, Massaro JM, Vasan RS. Predicting the 30-year risk of cardiovascular disease: the framingham heart study. Circulation. 2009;119(24):3078-3084. doi:10.1161/CIRCULATIONAHA.108.816694 19506114 PMC2748236

[15] Framingham heart study. Accessed May 20, 2023. https://www.framinghamheartstudy.org/

[16] Ying A, Arima H, Czernichow S, ; Blood Pressure Lowering Treatment Trialists’ Collaboration. Effects of blood pressure lowering on cardiovascular risk according to baseline body mass index: a meta-analysis of randomised trials. Lancet. 2015;385(9971):867-874. doi:10.1016/S0140-6736(14)61171-5 25468168

[17] Hsu CY, McCulloch CE, Darbinian J, Go AS, Iribarren C. Elevated blood pressure and risk of end-stage renal disease in subjects without baseline kidney disease. Arch Intern Med. 2005;165(8):923-928. doi:10.1001/archinte.165.8.923 15851645

[18] Hsu CY, Iribarren C, McCulloch CE, Darbinian J, Go AS. Risk factors for end-stage renal disease: 25-year follow-up. Arch Intern Med. 2009;169(4):342-350. doi:10.1001/archinternmed.2008.605 19237717 PMC2727643

[19] Pocock SJ, McCormack V, Gueyffier F, Boutitie F, Fagard RH, Boissel JP. A score for predicting risk of death from cardiovascular disease in adults with raised blood pressure, based on individual patient data from randomised controlled trials. BMJ. 2001;323(7304):75-81. doi:10.1136/bmj.323.7304.75 11451781 PMC34541

[20] Pharmacist billing/coding quick reference sheet for services provided in physician-based clinics. ASHP. Published June 2019. Accessed November 14, 2022. https://www.ashp.org/-/media/assets/ambulatory-care-practitioner/docs/billing-quick-reference-sheet.pdf

[21] Park C, Wang G, Ng BP, Fang J, Durthaler JM, Ayala C. The uses and expenses of antihypertensive medications among hypertensive adults. Res Social Adm Pharm. 2020;16(2):183-189. doi:10.1016/j.sapharm.2019.05.002 31085142

[22] Stagnitti MN. National health care expenses per person in the US civilian noninstitutionalized population, 2014. Agency for Healthcare Research and Quality. Accessed November 14, 2022. https://www.ncbi.nlm.nih.gov/books/NBK425794/28422469

[23] Annual data report. USRDS. Accessed May 20, 2023. https://adr.usrds.org/

[24] US consumer price index: medical care and commodities 2021. Statista. Accessed November 14, 2022. https://www.statista.com/statistics/187228/consumer-price-index-for-medical-care-services-in-the-us-since-1960/

[25] Sullivan PW, Lawrence WF, Ghushchyan V. A national catalog of preference-based scores for chronic conditions in the United States. Med Care. 2005;43(7):736-749. doi:10.1097/01.mlr.0000172050.67085.4f 15970790

[26] Estimated hypertension prevalence, treatment, and control among US adults. Centers for Disease Control and Prevention. Published March 22, 2021. Accessed November 14, 2022. https://millionhearts.hhs.gov/data-reports/hypertension-prevalence.html

[27] Lacruz ME, Kluttig A, Hartwig S, . Prevalence and incidence of hypertension in the general adult population: results of the CARLA-cohort study. Medicine (Baltimore). 2015;94(22):e952. doi:10.1097/MD.0000000000000952 26039136 PMC4616348

[28] Vaughan AS, Coronado F, Casper M, Loustalot F, Wright JS. County-level trends in hypertension-related cardiovascular disease mortality-United States, 2000 to 2019. J Am Heart Assoc. 2022;11(7):e024785. doi:10.1161/JAHA.121.024785 35301870 PMC9075476

[29] Anderegg MD, Gums TH, Uribe L, Coffey CS, James PA, Carter BL. Physician-pharmacist collaborative management: Narrowing the socioeconomic blood pressure gap. Hypertension. 2016;68(5):1314-1320. doi:10.1161/HYPERTENSIONAHA.116.08043 27600181 PMC5063695

[30] Carter BL, Coffey CS, Ardery G, . Cluster-randomized trial of a physician/pharmacist collaborative model to improve blood pressure control. Circ Cardiovasc Qual Outcomes. 2015;8(3):235-243. doi:10.1161/CIRCOUTCOMES.114.001283 25805647 PMC4618490

[31] Dixon DL, Sisson EM, Parod ED, . Pharmacist-physician collaborative care model and time to goal blood pressure in the uninsured population. J Clin Hypertens (Greenwich). 2017;(July):1-8. doi:10.1111/jch.13150 29237095 PMC8031164

[32] Doctor shortages are here—and they’ll get worse if we don’t act fast. American Medical Association. Accessed November 14, 2022. https://www.ama-assn.org/practice-management/sustainability/doctor-shortages-are-here-and-they-ll-get-worse-if-we-don-t-act

[33] Berenbrok LA, Tang S, Gabriel N, . Access to community pharmacies: a nationwide geographic information systems cross-sectional analysis. J Am Pharm Assoc (2003). 2022;62(6):1816-1822.e2. doi:10.1016/j.japh.2022.07.003 35965233

[34] Adams AJ, Weaver KK. The continuum of pharmacist prescriptive authority. Ann Pharmacother. 2016;50(9):778-784. doi:10.1177/1060028016653608 27307413

[35] McBane SE, Dopp AL, Abe A, ; American College of Clinical Pharmacy. Collaborative drug therapy management and comprehensive medication management-2015. Pharmacotherapy. 2015;35(4):e39-e50. doi:10.1002/phar.1563 25884536

[36] Levine DM, Linder JA, Landon BE. Characteristics of americans with primary care and changes over time, 2002-2015. JAMA Intern Med. 2020;180(3):463-466. doi:10.1001/jamainternmed.2019.6282 31841583 PMC6990950

[37] Dietrich E, Gums JG. Incident-to billing for pharmacists. J Manag Care Spec Pharm. 2018;24(12):1273-1276. doi:10.18553/jmcp.2018.24.12.1273 30479200 PMC10397947

