# Supplementary material for: Cost-Effectiveness of Pharmacist Prescribing for Managing Hypertension in the United States
Source: JAMA Netw Open. 2023 Nov 3;6(11):e2341408. doi: 10.1001/jamanetworkopen.2023.41408 (PMC10625044; doi:10.1001/jamanetworkopen.2023.41408)
Supplement: Supplement 1. — eTable 1. Assumed Patient Characteristics for Pharmacist Hypertension Intervention, Based on Observed Population in Tsuyuki et al10 Clinical Trial eTable 2. Model US Cost Inputs eFigure 1. Association Between Cumulative Cost Savings and QALY Benefit Across a Range of Hypothetical SBP Reductions Associated With the Pharmacist Intervention [file jamanetwopen-e2341408-s001.pdf]

Supplemental Online Content

Dixon DL, Johnston K, Patterson J, Marra C, Tsuyuki RT. Cost-effectiveness of pharmacist-prescribing for managing hypertension in the United States. *JAMA Netw Open*. 2023;6(10):e2341408. doi:10.1001/jamanetworkopen.2023.41408

eTable 1. Assumed Patient Characteristics for Pharmacist Hypertension Intervention, Based on Observed Population in Tsuyuki et al[10] Clinical Trial
eTable 2. Model US Cost Inputs
eFigure 1. Association Between Cumulative Cost Savings and QALY Benefit Across a Range of Hypothetical SBP Reductions Associated With the Pharmacist Intervention

This supplemental material has been provided by the authors to give readers additional information about their work.

© 2023 Dixon DL et al. *JAMA Network Open.*

eTable 1: Assumed patient characteristics for pharmacist hypertension intervention, based on observed population in Tsuyuki et al.[10] clinical trial

Characteristic	Value
Age (years)	63.5
Sex (% male)	48.8
Systolic blood pressure (mmHg)	149.5
Diastolic blood pressure (mmHg)	83.7
Treatment for hypertension (%)	77.8
Smoking (%)	16.5
Diabetes mellitus (%)	44.0
Body mass index (kg/m^2)	32.0

© 2023 Dixon DL et al. *JAMA Network Open.*

eTable 2: Model US Cost Inputs

Parameter	Value	Probabilistic	Source
**Cost of pharmacist intervention**			
Cost per consultation	$23.10		CPT level 1
Number of consultations (Year 1)	6		Monthly until controlled, followed by quarterly
Number of consultations (Year 2+)	4		
**Cost of stroke**			Dehmer et al. AHRQ/MEPS
Year 1	$18,192	Gamma (25,921)	
Year 2+	$5,389	Gamma (25, 273)	
**Cost per year of heart failure**			Dehmer et al. AHRQ/MEPS
Year 1	$30,068	Gamma (25, 1521)	
Year 2+	$11,583	Gamma (25, 586)	
**Cost per year of angina**			Dehmer et al. AHRQ/MEPS
Year 1	$24,290	Gamma (25,1229)	
Year 2+	$4,262	Gamma (25,216)	
**Cost of myocardial infarction**			Dehmer et al. AHRQ/MEPS
Year 1	$37,095	Gamma (25, 1877)	

© 2023 Dixon DL et al. *JAMA Network Open.*

Year 2+	$2,490	Gamma (25, 126)	
**Cost per year of end-stage kidney disease**	$78,199	Gamma (25, 3378)	USRDS
**Cost of background medical costs**	$5,531	Gamma (25, 266)	AHRQ
AHRQ, Agency for Healthcare Research and Quality; CPT, Current Procedural Terminology; MEPS, Medication Expenditures Panel Survey; USRDS, United States Renal Data System			

© 2023 Dixon DL et al. *JAMA Network Open.*

eFigure 1: Relationship between cumulative cost savings and QALY benefit across a range of hypothetical SBP reductions associated with the pharmacist intervention

QALY=quality-adjusted life year

© 2023 Dixon DL et al. *JAMA Network Open.*

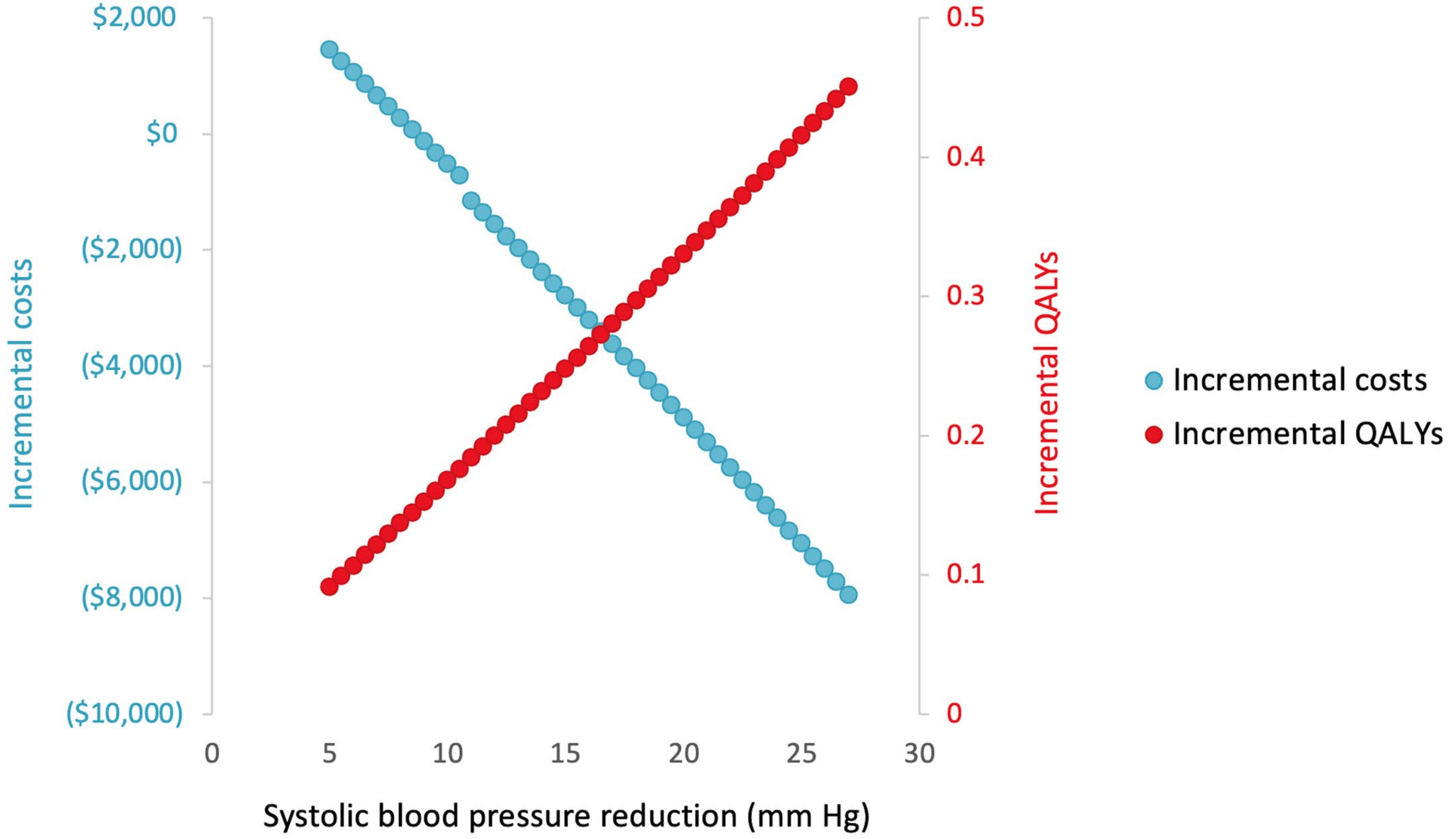

© 2023 Dixon DL et al. *JAMA Network Open.*
