# Supplementary material for: Cost-Effectiveness of Pharmacist Prescribing for Managing Hypertension in the United States
Source: JAMA Netw Open. 2023 Nov 3;6(11):e2341408. doi: 10.1001/jamanetworkopen.2023.41408 (PMC10625044; doi:10.1001/jamanetworkopen.2023.41408)
Supplement: Supplement 2. — Data Sharing Statement [file jamanetwopen-e2341408-s002.pdf]

Data Sharing Statement

Dixon. Cost-Effectiveness of Pharmacist Prescribing for Managing Hypertension in the United States. *JAMA Netw Open*. Published November 03, 2023.
doi:10.1001/jamanetworkopen.2023.41408

Data
Data available: No
